# Real-life survey of pitfalls and successes of precision medicine in genetic epilepsies

**DOI:** 10.1136/jnnp-2020-325932

**Published:** 2021-04-26

**Authors:** Simona Balestrini, Daniela Chiarello, Maria Gogou, Katri Silvennoinen, Clinda Puvirajasinghe, Wendy D Jones, Philipp Reif, Karl Martin Klein, Felix Rosenow, Yvonne G Weber, Holger Lerche, Susanne Schubert-Bast, Ingo Borggraefe, Antonietta Coppola, Serena Troisi, Rikke S Møller, Antonella Riva, Pasquale Striano, Federico Zara, Cheryl Hemingway, Carla Marini, Anna Rosati, Davide Mei, Martino Montomoli, Renzo Guerrini, J Helen Cross, Sanjay M Sisodiya

**Affiliations:** 1 Department of Clinical and Experimental Epilepsy, UCL Queen Square Institute of Neurology, London, and Chalfont Centre for Epilepsy, Gerrard Cross, UK; 2 Neurology Unit and Neurogenetics Laboratories, Meyer Children Hospital, Florence, Italy; 3 Institute of Child Health, University College of London (UCL) Great Ormond Street NIHR BRC, London, UK; 4 Great Ormond Street Hospital for Children, London, UK; 5 Epilepsy Center Frankfurt Rhine-Main University of Frankfurt, University of Frankfurt, Frankfurt Rhine Main, Germany; 6 Department of Neurology, University Hospital Frankfurt and LOEWE Center for Personalized Translational Epilepsy Research (CePTER) Goethe-University Frankfurt, Frankfurt am Main, Germany; 7 Epilepsy Center Hessen and Department of Neurology, Philipps-University, Marburg, Germany; 8 Departments of Clinical Neurosciences, Medical Genetics and Community Health Sciences, Hotchkiss Brain Institute & Alberta Children’s Hospital Research Institute, Cumming School of Medicine, University of Calgary, Calgary, Alberta, Canada; 9 Department of Neurology and Epileptology, University of Tübingen, Tubingen, Germany; 10 Department of Epileptology and Neurology, University of Aachen, Aachen, Germany; 11 Department of Pediatric Neurology, Dr von Haunerschen Kinderspital, University of Munich, Munich, Germany; 12 Department of Neuroscience, Reproductive and Odontostomatological Sciences, Epilepsy Centre, Federico II University, Naples, Italy; 13 The Danish Epilepsy Centre Filadelfia, Dianalund, and Institute for Regional Health Services Research, University of Southern Denmark, Odense, Denmark; 14 Department of Neurosciences, Rehabilitation, Ophtalmology, Genetics, Maternal and Child Health, University of Genova, Genova, Italy; 15 IRCCS ‘G. Gaslini’ Institute, Genova, Italy; 16 Child Neurology and Psychiatric Unit, Salesi Children's Hospital, Ancona, Italy

## Abstract

**Objective:**

The term ‘precision medicine’ describes a rational treatment strategy tailored to one person that reverses or modifies the disease pathophysiology. In epilepsy, single case and small cohort reports document nascent precision medicine strategies in specific genetic epilepsies. The aim of this multicentre observational study was to investigate the deeper complexity of precision medicine in epilepsy.

**Methods:**

A systematic survey of patients with epilepsy with a molecular genetic diagnosis was conducted in six tertiary epilepsy centres including children and adults. A standardised questionnaire was used for data collection, including genetic findings and impact on clinical and therapeutic management.

**Results:**

We included 293 patients with genetic epilepsies, 137 children and 156 adults, 162 females and 131 males. Treatment changes were undertaken because of the genetic findings in 94 patients (32%), including rational precision medicine treatment and/or a treatment change prompted by the genetic diagnosis, but not directly related to known pathophysiological mechanisms. There was a rational precision medicine treatment for 56 patients (19%), and this was tried in 33/56 (59%) and was successful (ie, >50% seizure reduction) in 10/33 (30%) patients. In 73/293 (25%) patients there was a treatment change prompted by the genetic diagnosis, but not directly related to known pathophysiological mechanisms, and this was successful in 24/73 (33%).

**Significance:**

Our survey of clinical practice in specialised epilepsy centres shows high variability of clinical outcomes following the identification of a genetic cause for an epilepsy. Meaningful change in the treatment paradigm after genetic testing is not yet possible for many people with epilepsy. This systematic survey provides an overview of the current application of precision medicine in the epilepsies, and suggests the adoption of a more considered approach.

## Introduction

In the fifth century BC, Hippocrates stated that ‘human beings are innately (genetically) different from one another, and this individuality affects both their predisposition/susceptibility to disease and their response to therapeutics’. This was the first documentation of the central principle of ‘precision medicine’ (PM), thousands of years ago.^1^ PM is a treatment approach in which disease treatment and prevention is tailored to individual variability in genes, environment and lifestyle for each person.^2^ Best clinical practice has always been to provide PM, for instance, by taking into account all available demographic, clinical and instrumental information of each patient, typically within an intuitive Bayesian framework integrating serial data collection into a global evaluation of patient diagnosis and treatment response.^3^ Now, genomic data add another layer of information to this framework, with the promise of even more customised diagnosis and treatment.

An increasing number of syndromes has been discovered in which all or much of the clinical picture is attributed to variation in a single gene. This in turn has allowed a better understanding of disease biology, and has facilitated the identification of molecular targets for precision deployment of new and repurposed compounds in the treatment of the epilepsies. There is some evidence of effective PM strategies in epilepsy in relation to its specific underlying cause.^4–13^ However, some genetic epilepsies have proven resistant to current treatments and it is hoped that as work in this field continues, more genetic epilepsies will have targeted treatment available.^14^

However, some caution is needed. There is a gap between the much-promoted idea of PM in the genetic literature in the epilepsies, and progress for the majority of people with epilepsy. Cases with genetic diagnoses but no ensuing treatment implications, naturally, largely go unpublished: such bias risks giving an unduly optimistic impression of the applicability or simplicity of PM.

Recent exome sequencing studies in children with drug-resistant epilepsies reported a diagnostic yield of ~30% with positive impact on clinical management or treatment following genetic diagnosis only in a minority of cases.^15 16^

While identifying a genetic aetiology is undoubtedly beneficial at many levels in epilepsy,^17^ the implications for treatment remain limited, and there is a gap in understanding why some putative PM treatments fail, lead to adverse effects or are not used.^18^ We aimed to assess the impact of genetics in the current PM approach to epilepsy, focusing on the variability of outcomes following genetic diagnoses in a multicentre cohort including both children and adults.

A multicentre systematic survey of patients with a molecular genetic diagnosis of epilepsy was conducted at the National Hospital for Neurology and Neurosurgery (NHNN, London, UK (n=146)), Great Ormond Street Hospital (GOSH, London, UK) (n=93), Children's Hospital A. Meyer (Florence, Italy) (n=28), Federico II University Hospital (Naples, Italy) (n=13), ‘Giannina Gaslini’ Institute (Genova, Italy) (n=11), and Institute for Clinical Brain Research (Tubingen, Germany) (n=2). At NHNN and GOSH, the study was registered as a service evaluation, and the requirement of consent was waived by the local ethics committees. All cases from other centres had consent/assent for data sharing (either from patients or from parents or legal guardians). For all centres, consecutive children (<18 years) and adults (≥18 years) seen in the epilepsy clinic with last follow-up between 2007 and 2019 were identified by reviewing clinical and research databases.

## Materials and methods

It is important to note that this was an unblinded, retrospective observational study, and not a trial (randomised controlled or other): therefore, genetic testing strategies varied from centre to centre and case to case as well as over time. We aimed to report actual current clinical practice across several centres, addressing cases with molecular genetic diagnoses already previously accepted by the local clinical team.

**Figure 1:**
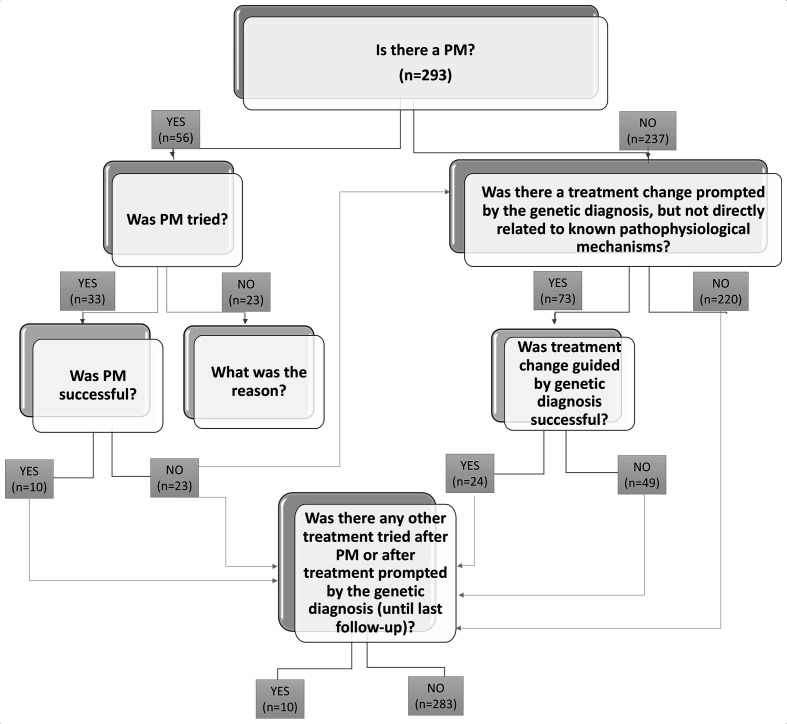
Algorithm illustrating clinical management and outcome following the discovery of genetic aetiology.

Inclusion criteria were as follows: people with epilepsy and an underlying genetic condition considered to be causative of the epilepsy. A standardised questionnaire was used for collection of data, including clinical history, details of genetic testing and impact on clinical management (https://forms.office.com/Pages/ResponsePage.aspx?id=_oivH5ipW0yTySEKEdmlwsIxMcWkeRpJskQT7bkOQRFUNUI1NVZCVkxSVkQ4TEhZNElRU0tIV0I2TC4u). To evaluate the latter, we used a flow chart illustrating clinical management and outcome following the discovery of genetic aetiology (figure 1). Seizures and epilepsy were classified according to the latest ILAE definitions.^19^ Classification of treatment was based on existing literature and PM mechanisms were established for the following genes *ARG1, DEDPC5, CHRNA4, GAMT, GRIN2A, KCNA2, KCNH2, KCNQ2, MTOR, SCN1A, SCN8A, SCN2A, SLC2A1, TSC1, TSC2*, according to the relevant literature.^4–13 20–22^,

Successful treatment was defined as a reduction in seizure frequency >50%. Cognitive outcome was included when available, but this was mostly based on the clinician’s subjective impression or subjective report of parents/carers and rarely referred to formal neuropsychometric assessment. Clinicians were asked to determine whether there was an improvement in the quality of life following the genetic diagnosis; however, this was not included in the statistical analysis as it was only a subjective evaluation. For single-nucleotide variants (SNVs), the American College of Medical Genetics and Genomics (ACMG) guidelines were used for variant interpretation^23^ by a multidisciplinary team including clinical scientists, clinical geneticists and paediatric or adult neurologists. Chromosomal imbalances, copy number variants (CNVs) and repeat expansions were deemed pathogenic according to the phenotypic presentation and overall assessment by the responsible clinicians. Exclusion criteria were as follows: cases with variants identified in genes that cause Mendelian disorders and classified as ‘likely benign’ or ‘benign’ according to the ACMG criteria,^23^ and cases with no details on treatment changes after the genetic diagnosis. We included cases with variants classified as ‘of uncertain significance’,^23^ providing that they were considered to be causative of, or contributing to, the epilepsy by the respective clinicians, even if this was not formally confirmed in a multidisciplinary discussion.

### Statistical analysis

The Pearson χ^2^ or Fisher’s exact tests, as appropriate, for binary or categorical variables, and *t*-test for continuous variables, were used to analyse the association of outcome following the identification of the underlying genetic abnormality and clinical and other genetic factors. No variable had more than 5% missing data: missing data were therefore omitted, with no other correction or interpolation undertaken. The threshold for statistical significance was set at p<0.05. Data analysis was performed using the Stata/IC V.11.1 Statistical package.

## Results

### Clinical and genetic assessment

In total 293 patients (162 females, 131 males; 137 children, 156 adults) with a confirmed genetic aetiology for their epilepsy were included in the main analysis. Clinical details are summarised in table 1. The genetic diagnosis was achieved through various methods including single gene capillary sequencing, array-comparative genomic hybridisation, karyotype analysis, whole exome sequencing gene panels, and more broadly, whole exome and genome sequencing (the latter often as part of research studies). When an initially detected genetic abnormality did not explain the phenotype at all or completely, further genetic investigations were often undertaken, according to local strategies. We note that our study was not to address the relative merits of different types of genetic testing, but to determine outcome related to treatment following a genetic diagnosis that was considered by the local clinical team to be the cause of an individual patient’s epilepsy. The genetic abnormalities considered causative of the epilepsy included SNVs (n=248, 85% of patients), chromosomal imbalances (n=21, 7%), CNVs (n=17, 6%), repeat expansion (n=5, 2%), SNV plus CNV (n=2, 1%). Only five gene variants were functionally tested: one missense variant in the *KCNA2* gene caused gain-of-function, and led to a treatment trial with 4-aminopyridine^22–24^; one missense variant in the *DNM1* gene with a dominant-negative mechanism^25^; one missense variant in the *SCN2A* gene caused gain-of-function and led to introduction of carbamazepine; one missense variant in the *SCN8A* gene caused gain-of-function and led to introduction of carbamazepine; one truncating variant in the *SCN1A* gene caused loss-of-function but did not lead to any treatment changes.

**Table 1:** Summary of clinical details of the 293 patients included in the multicentre systematic survey

Gender		162 females, 131 males
Mean age at last follow-up		22 years, SD 16, median 19, range 8 months to 69 years
Patients deceased		17 (6%)
Family history of epilepsy or febrile seizures		131 (45%)
Type of putative causal genetic abnormality	SNV	248 (85%)
	CNV	17 (6%)
	SNV+CNV	2 (1%)
	Chromosomal imbalance	21 (7%)
	Repeat expansion	5 (2%)
Mean age at seizure onset		4 years, SD 5, median 1, range 1 day to 30 years
Mean age at clinical diagnosis of the epilepsy syndrome		11 years, SD 13, median 5, range antenatal to 58 years
Mean age at genetic diagnosis		16 years, SD 15, median 13, range 1 day to 63 years
Mean interval from clinical to genetic diagnosis		5 years, SD 10, median 0, range 4 years earlie to 52 years afterwards
Developmental delay or regression		216 patients (75%)
Mean age at onset of developmental slowing		16 months, SD 16, median 10, range from birth to 96 months
History of febrile seizures		106 (36%)
Drug-resistant epilepsy		240 (82%)

CI, chromosomal imbalance; CNV, copy number variant; SNV, single-nucleotide variant.

At the time of the genetic testing, encephalopathy was reported in 208 patients (71%), either developmental and epileptic (n=127, 61%), epileptic (n=68, 33%) or developmental (n=13, 6%). Dravet syndrome (DS), with an underlying variant in *SCN1A*, was present in 70 (24%) cases. Comorbidities were reported in 257 patients (88%).

### Impact of genetic diagnosis

Most patients had received multiple antiseizure medications prior to genetic diagnosis, with a median number of antiseizure medications tried before the genetic diagnosis of five (SD 4, range 0–19). Other treatment for their epilepsy had included: ketogenic diet or other dietary treatment in 66 patients (23%), vagus nerve stimulation in 23 patients (8%) and neurosurgical resection, disconnection or callosotomy in 20 patients (7%). Surgical treatment, performed either before or after the genetic diagnosis, was successful in 9/20 patients (45%), leading to seizure freedom (3/20) or improved seizure control (6/20). In 78 patients (27%), there was a history of seizure worsening (based on clinician evaluation) associated with the use of one or more antiseizure medications before the molecular genetic diagnosis. Following the genetic diagnosis, in 101 patients (35%) further diagnostic assessment was arranged (eg, cardiac assessment or any other test performed as a consequence of the genetic diagnosis), and in 94 patients (32%) medication changes were undertaken because of the genetic findings. Overall an improvement in quality of life following definitive molecular genetic diagnosis was reported by the clinician for 114 patients (39%).

Following the scheme for PM illustrated in figure 1, there was an existing established PM treatment for 56 patients (19%). Of these, the established PM was tried in 33/56 (59%) after genetic diagnosis, and was reported as successful in 10/33 (30%) patients (table 2). Reasons for not trying the established PM treatment included: already seizure-free or seizure control deemed acceptable to the family and clinician when the genetic diagnosis was made (n=9), other treatment effective (n=5), recent molecular diagnosis or no further follow-up after genetic diagnosis (n=3), parents declined (n=2), patient deceased (n=1), difficulties in accessing funding for treatment (n=1) or unknown (2). The outcome of PM trial was not successful in 23/33 cases (70%): in two cases PM (withdrawal of sodium channel blockers in *SCN1A*-related DS) was not associated with significant seizure improvement, but improvement of cognitive function was noted; in one case PM (withdrawal of oxcarbazepine in *SCN1A*-related DS) was associated with >50% reduction in seizure frequency, but the improvement was not sustained; in four cases PM (trial of everolimus in *TSC2*-related Tuberous Sclerosis Complex, use of phenytoin in presumed gain-of-function *SCN2A*-related epileptic encephalopathy, withdrawal of phenytoin in *SCN1A*-related DS, trial of 4-aminopyridine in gain-of-function *KCNA2-*related epileptic encephalopathy) was associated with clinical deterioration and seizure worsening; in one case PM (ketogenic diet in GLUT1 deficiency syndrome) was effective but not tolerated. In 7/33 (21%) some improvement in seizure frequency after a change to PM was reported, but <50%. In 8/33 (24%), no clinical effect was reported (table 3).

**Table 2:** List of the 10 patients where a PM strategy was tried and was successful (>50% seizure reduction)

Gene variant and details	Age at clinical diagnosis (years)	Age at genetic diagnosis (years)	No of ASMs tried before genetic diagnosis/other non-medical treatment	Changes of treatment following genetic diagnosis	Age at last follow-up (years)	Diagnostic assessment following genetic diagnosis	Previous ASMs worsening seizure control
Homozygous *ARG1* (c.93del, p.Arg32fs), pathogenic	Unknown	29	1	Low-protein diet	33	None	None
Heterozygous *DEPDC5* (c.727C>T, p.Arg243*), pathogenic	11	26	Nine plus cortical resection	Introduction of ketogenic diet	30	ECG	Oxcarbazepine
Homozygous *GAMT* (c.327G>A, p.Lys109=), likely pathogenic	25	26	Seven plus callosotomy	Introduction of creatine supplements, withdrawal of sodium valproate	31	Metabolic assessment	None
Heterozygous *KCNH2* (c.3125_3135dupTGGATGCCCTC, p.Gln1046Trpfs*15), pathogenic	2	0.4	3	Beta-blocker therapy (nadolol)	3	Regular cardiac assessment surveillance, QT interval measurement	None
Heterozygous *SCN1A* (c. 602+1G>A, p.(?)), pathogenic	7	6	8	Withdrawal of carbamazepine, introduction of stiripentol	21	None	Carbamazepine
Heterozygous *SCN1A* (c.992delT, p.Leu331X), pathogenic	58	59	5	Withdrawal of primidone and carbamazepine, introduction of levetiracetam	69	None	None
Heterozygous *SCN1A* (c.1624C>T, p.Arg542*), pathogenic	27	27	15	Withdrawal of lamotrigine, increase of sodium valproate	31	Speech and language assessment for dysphagia	Oxcarbazepine, tiagabine, lamotrigine
Heterozygous *SCN1A* (c.2729A>C, p.Gln910Pro), likely pathogenic	48	48	11	Withdrawal of carbamazepine	58	None	Lamotrigine, phenobarbitone
Heterozygous *SCN1A* (c.2792G>A, p.Arg931His), likely pathogenic	41	43	10	Introduction of stiripentol and clobazam, withdrawal of carbamazepine	53	None	Topiramate, levetiracetam, zonisamide, pregabalin, lacosamide, clobazam, carbamazepine, sodium valproate, stiripentol, lamotrigine
Heterozygous *SLC2A1* (c.823G>A, p.Ala275Thr), pathogenic	11	6	1	Introduction of ketogenic diet	12	Regular cognitive assessment	None

ASM, antiseizure medication; PM, precision medicine.

**Table 3:** List of the 23 patients where a PM strategy was tried and was not successful (<50% seizure reduction)

Gene variant and details	Age at clinical diagnosis (years)	Age at genetic diagnosis (years)	No of ASMs tried before genetic diagnosis/other non-medical treatment	Changes of treatment following genetic diagnosis, and details of outcome	Age at last follow-up (years)	Diagnostic assessment following genetic diagnosis	Previous ASMs worsening seizure control
Heterozygous *CHRNA4* (c.851C>T, p.Ser284Leu), likely pathogenic	16	22	7	Introduction of nicotine patches and zonisamide, both ineffective	24	None	None
Heterozygous *CHRNA4* (c.868C>A, p.Leu290Met), uncertain significance	24	24	7	Introduction of galantamine, ineffective	33	None	None
Heterozygous *KCNA2* (c.894G>T, p.Leu298Phe), likely pathogenic	2	36	13	Introduction of 4-aminopyridine, seizure deterioration	41	ECG	None
Heterozygous *SCN1A* (c.264+3 del), uncertain significance	38	38	14 plus VNS	Withdrawal of carbamazepine, introduction of cannabidiol and zonisamide, ineffective	51	None	None
Heterozygous *SCN1A* (c.603-2A>G, p.(?)), pathogenic	20	20	13 plus VNS	Withdrawal of carbamazepine, introduction of cannabidiol and stiripentol, seizure reduction <50%	31	None	Lamotrigine
Hterozygous *SCN1A* (c.1754dup), pathogenic	29	25	10	Withdrawal of carbamazepine, seizure control stable, improved cognition	29	ECG	None
Heterozygous *SCN1A* (c.2435C>T, p.Thr812Ile), uncertain significance	15	15	Five plus callosotomy and VNS	Withdrawal of phenytoin, seizure worsening	20	Unknown	Unknown
Heterozygous *SCN1A* (c.2834_2847del, p(Phe945Trpfs*47), pathogenic	31	31	18 plus VNS	Withdrawal of carbamazepine, seizure reduction <50%	38	None	Lamotrigine
Heterozygous *SCN1A* (c.2836C>T, p.Arg946Cys), likely pathogenic	17	17	Eight plus KD and VNS	Withdrawal of lacosamide and re-introduction of valproate, ineffective	18	None	None
Heterozygous *SCN1A* (c.3797A>C, p.Glu1266Ala), likely pathogenic	16	26	Ten plus KD	Withdrawal of carbamazepine, introduction of valproate and cannabidiol, seizure reduction <50%	38	None	Lamotrigine
Heterozygous *SCN1A* (c.4205_4208del, p.Arg1402Metfs*9), pathogenic	4	17	14 plus KD	Withdrawal of phenobarbitone, introduction of stiripentol and zonisamide, seizure reduction <50%	29	None	Carbamazepine, lamotrigine
Heterozygous *SCN1A* (c.4384T>C; p.Tyr1462His), likely pathogenic	14	18	15 plus VNS	Introduction of clobazam, withdrawal of primidone and phenobarbitone, seizure control stable, cognition improvement	39	None	Lamotrigine, levetiracetam
Heterozygous *SCN1A* (c.4396_4372dupCTGT; p.Tyr1458Serfs), pathogenic	27	28	9	Withdrawal of oxcarbazepine, initial >50% seizure reduction but not sustained	37	None	None
Heterozygous *SCN1A* (c.4568T>C p.I1523T), likely pathogenic	36	40	17	Withdrawal of lamotrigine, introduction of stiripentol, ineffective	47	None	None
Heterozygous *SCN1A* (c.5092G>T, p.Glu1698*), likely pathogenic	1	20	Ten plus KD	Withdrawal of oxcarbazepine, introduction of stiripentol and cannabidiol, seizure reduction <50%	25	ECG	None
Heterozygous *SCN1A* (c.5468T>C, p.Met1823Thr), uncertain significance	Unknown	54	12	Withdrawal of oxcarbazepine, introduction of clobazam, seizure reduction <50%	61	None	Carbamazepine, phenytoin
Heterozygous *SCN2A* (c.4037T>A, p.Ile1346Asn), uncertain significance	0.1	17	Six plus KD	Introduction of carbamazepine, seizure reduction <50%	23	ECG	None
Heterozygous *SCN2A* (c.4949T>C, p.Leu1650Pro), likely pathogenic	1	14	7	Introduction of phenytoin, severe seizure deterioration	19	Cardiac assessment	None
Heterozygous *SCN8A* (c.2287A>G, p.I763V), likely pathogenic	0.5	20	5	Introduction of phenytoin, increased dose of carbamazepine, ineffective	25	None	None
Heterozygous *SCN8A* (c.2921C>G, p.Ala974Gly), pathogenic	11	10	12 plus KD	Introduction of phenytoin, ineffective	12	Cardiac assessment	None
Heterozygous *SLC2A1* (c.1437C>T, p.Pro479=), uncertain significance	8	9	Six plus KD	Introduction of KD, effective but not tolerated	15	None	None
Heterozygous *TSC2* (c.3671_3674del, p.Asn1224Thrfs*100), pathogenic	0.4	Unknown	8	Introduction of everolimus, clinical deterioration including seizure worsening	26	Multidisciplinary assessment including neurological, renal and cardiac surveillance	None
Heterozygous *TSC2* (c.4639_4642del, p.Val1547Cysfs*28), pathogenic	1	28	Nine plus cortical resection	Introduction of everolimus, ineffective	32	Multidisciplinary assessment including neurological, renal and cardiac surveillance	None

ASM, antiseizure medication; KD, ketogenic diet; PM, precision medicine; VNS, vagus nerve stimulation.

In 73/293 (25%) patients, there was a treatment change prompted by the genetic diagnosis (16 of them also had PM trialled), but not directly related to known pathophysiological mechanisms (eg, valproate and stiripentol in *SCN1A*-related epilepsies). This was reported as successful in 24/73 (33%).

At last follow-up, the overall clinical condition compared with before genetic diagnosis, was reported as stable in 177 patients (60%), improved in 98 (33%) and worse in 18 (6%).

PM was available for epilepsies caused by SNVs (n=55) or CNVs (n=1), not for the other causal genetic abnormalities (SNV+CNV, chromosomal imbalance or repeat expansion disorders) (p=0.027, Fisher’s exact test). Successful PM was associated with improved overall condition at the last follow-up (p=0.013, Fisher’s exact test). Similarly, other treatment change prompted by the genetic diagnosis but not directly related to a PM mechanism was associated with improved outcome (p=0.001, Fisher’s exact test). Patients with improved outcome at the last follow-up had had a genetic diagnosis at a significantly younger age than patients with stable or worse outcomes (mean age at genetic test 13 vs 18 years, t-test p=0.021), independent of PM or other treatment changes.

ACMG classification was applicable for 217/248 SNVs, and included 114 pathogenic (53%), 71 likely pathogenic (33%) and 32 of uncertain significance (15%). Some molecular genetic diagnoses were made before ACMG guidelines were available, and were then made according to best local practice at the time. Analysis of the historical management (that had been undertaken according to contemporary best practice but now classified by ACMG class) of the variant did not show any difference between class 4 or class 5 variants versus class 3 variants for diagnostic management (eg, additional investigation prompted by the genetic findings) or treatment changes following identification of the genetic abnormality (table 4). We also observed that all patients with successful PM strategies had either likely pathogenic (3, 30%) or pathogenic variants (7, 70%), while patients with epilepsies where PM was not successful had also variants of uncertain significance (6, 26%) in addition to likely pathogenic (9, 39%) or pathogenic variants (8, 25%). The variant distribution according to PM outcome was not significant (p=0.120, Fisher’s exact test).

**Table 4:** Outcome at last follow-up and its association with underlying genetic abnormality and medication changes following the genetic diagnosis

Outcome at last follow-up (compared with before genetic diagnosis), n (%)	Successful PM treatment tried and successful*, n (%)	Treatment change prompted by the genetic diagnosis, but not directly related to known pathophysiological mechanisms, successful*, n (%)	Genetic abnormality, n (%)	ACMG classification >3,^23^ when applicable, n (%)
A.Improved, 98 (34)	7 (70)	16 (64)	85 SNV (87%) 6 CNV (6%) 6 CI (6%) one repeat expansion (1%)	56 (84%)
B.Stable, 177 (60)	3 (30)	8 (32)	147 SNV (84%) 10 CNV (6%) 1 SNV+CNV (1%) 15 CI (9%) two repeat expansion (1%)	121 (88%)
C.Worse, 18 (6)	0 (0)	1 (4)	14 SNV (78%) 1 CNV (6%) 1 SNV+CNV (6%) two repeat expansion (11%)	7 (64%)

*Successful treatment was defined as reduction >50% in seizure frequency.

ACMG, American College of Medical Genetics and Genomics; CI, chromosomal imbalance; CNV, copy number variant; PM, precision medicine; SNV, single-nucleotide variant.

## Discussion

Our analysis shows a broad variety of outcomes following identification of a plausible underlying genetic cause for an individual’s epilepsy.

The best outcome related to PM, when altered management strategies were available and proved successful, was achieved in only 3% of the entire cohort (10/293), demonstrating the currently limited reach of PM in the epilepsies in day-to-day practice, in the absence of a systematic programme for PM usage, even in tertiary referral centres. Established PM strategies existed for 19% of patients, were tried in only 11%, and, when tried, were not successful in 23/33 patients (70%): the majority (82%) of our cohort remained in the drug-resistant state despite the existence of putative PM strategies for some patients.

Among the 41% of patients where PM recognised treatments indicated by the genetic finding were not tried, there were some instances where there were difficulties in accessing funding for specific therapies, such as everolimus in tuberous sclerosis. This represents a set of issues frequently encountered in rare genetic conditions, because in the context of local regulations and lack of robust evidence (eg, lack of randomised controlled trials) obtaining approval or funding for off-licence treatments can prove difficult. These issues should be addressed, especially when there is more than anecdotal evidence that PM treatments may be effective and could also reduce the health cost burden.^7 26^ In many (n=14/23) of the 41% of patients where there was an existing PM strategy that was not tried, this was because seizure control was already satisfactory or patients were seizure-free on alternative treatment, established before the genetic findings.

In our cohort, variables that were significantly associated with improved clinical condition at the last follow-up included any treatment change prompted by the genetic diagnosis, either PM or other not directly related to a PM mechanism, emphasising how treatment guided by the underlying genetic abnormality can drive clinical improvement. Our findings also highlight that a genetic diagnosis should be pursued at any age (in our cohort, more adults had successful PM treatment than children), although the chance of improving outcome may fall over time (we found that lower age at genetic test was associated with better outcome): we acknowledge the small numbers of patients and outcomes on which these inferences are made.

Despite a lack of PM therapies, or these being unsuccessful, improvement in the overall clinical condition compared with before genetic diagnosis was reported in 33%. This was significantly associated with treatment changes prompted by the genetic diagnosis, but not directly related to known pathophysiological mechanisms, that were undertaken in 25% of patients and were successful in 8% of the entire cohort. Other factors that may contribute to clinical improvement following genetic diagnosis include amelioration of aspects other than seizures such as cognition, arrangement of further diagnostic assessment as a specific genetic diagnosis may implicate other organ involvement and inform prognosis, reduction of seizure frequency <50% but still with relevant impact on daily life.

We note that a minority of individuals (20/293) in our cohort had surgical treatment for the epilepsy (including callosotomy, resective surgery or disconnection), and this was successful in 45% of operated cases. There is still little evidence on how genetic findings may guide the selection of surgical candidates,^27^ but certainly an underlying genetic condition should not exclude a priori consideration for surgical treatment.

In our cohort, only five gene variants were functionally tested and this may contribute to explanation of the failure of the PM approach even when there was a recognised or predicted appropriate epilepsy treatment, as the actual functional consequence of a genetic variant is often presumed rather than determined empirically. There is growing evidence that experimental studies of the functional consequences of specific variants can explain phenotypic variability and severity.^28 29^ There are genes with established evidence of gain-or loss-of-function variants affecting phenotypic presentation and treatment response such as *SCN2A* and *SCN8A*.^9 10^ Patients with the few variants functionally tested in our study did not show successful clinical response to the appropriate PM (table 3). It is worth emphasising that in vitro experiments examining isolated functional consequences of a given genetic variant, for example, a specific ion channel gene variant, do not take into account various other influences (eg, epigenetics, environmental factors), and may not always prove relevant.

To help deal with multiple variants emerging from next-generation sequencing methods, systematic criteria for pathogenicity have been introduced, though previously local best practices would also have been used to assess pathogenicity. When we used the ACMG criteria to classify the variants, we observed no difference in the impact on clinical and therapeutic management between variants of uncertain significance (class 3) and variants that were likely pathogenic or pathogenic (class 4 or class 5). However, we note that there were only likely pathogenic or pathogenic variants in the group of patients with successful PM, while 26% of the patients with PM failure had variants of uncertain significance. Our cohort is too small to draw definitive inferences from this observation; however, variant classification, as a surrogate for variant pathogenicity, may represent a contributing factor to PM response. We note that some of the pieces of evidence required to assess pathogenicity can be difficult to obtain in patients with epilepsy, such as clinical correlation between implicated gene and clinical features (eg, dysmorphism is reasonably rare in epileptic encephalopathies, lack of gene-specific phenotypes), or absence of segregation data for adult patients when parents might be unavailable. The well-known phenotypic variability associated with genetic variants may also contribute to the observed variability in outcomes.^30–32^

With whole exome or genome sequencing, there is often a set of potentially causal variants identified needing discussion in a multidisciplinary setting to assess their significance for the patient’s phenotype. This is the case even if a non-genome-wide approach (eg, virtual epilepsy gene panel) has been applied. Filters such as allele frequency, inheritance status of the variant, predicted protein consequence, and tolerance to variation of the implicated gene may be employed to select the best candidate. However, all these filters still carry a degree of uncertainty and often selection remains arbitrary to some extent. Also, due to genetic pleiotropy, different variants within the same gene may cause distinct clinical disorders and therefore presenting different response to treatment. When PM is not successful, and if the genetic diagnosis was made through whole exome or genome sequencing, a revision of the previous variant selection strategy might be appropriate. Failure of the PM approach may not only lie in variant selection, but also in the genomic background including modifier variants in other genes, epigenetic and other regulatory processes, gene expression, environmental factors, lack of functional analysis, and perhaps other, currently unknown, mechanisms. A contemporary PM approach should include the search for precision explanation for failure of PM treatments: this should improve our knowledge of disease biology in specific individuals and differentiate the PM paradigm from the current system of drug choice which is based on the syndromic classification and can often be a trial-and-error process.^33^ However, the systematic use of exome sequencing in people with epilepsy is not currently possible in many centres due to time and cost implications, while gene panels are often faster and cheaper to apply and therefore most commonly used in typical practice. We also aimed to illustrate the variability of actual current clinical practice across several centres over the last 20 years, including patients diagnosed with genetic abnormalities preceding the advent of next generation sequencing.

Moreover, unpredicted responses to PM may also reflect phenotypic extension to the already accepted disease spectrum, a phenomenon seen in many genetic encephalopathies.^34^ This emphasises the need for a better understanding of pathophysiology in epilepsy—the need to understand in a full PM approach why an unexpected treatment is effective. At present, we do not have a comprehensive approach addressing the many potential layers contributing to the complexity of the PM approach. A partial solution may be to adopt a systems-level approach: one application based on transcriptomic analysis predicted valproate to preferentially restore to normality the disrupted gene expression network in epilepsy.^35^ However, valproate is not effective in many epileptic encephalopathies,^4^ including in DS where it represents one of the recommended treatment strategies,^36^ suggesting that other factors are also likely to play a role in treatment response.

Limitations of our study include lack of information on the duration of benefits derived from the medication changes, lack of a systematic analysis of treatment efficacy (eg, successful treatment may refer to a broader concept including impact on cognition, and other phenotypic aspects), side effects and of impacts on comorbidities, subjectivity of the treating clinician’s evaluation of changes in seizure control and/or quality of life. We acknowledge the bias that all of the centres included in our study are tertiary services highly specialised in genetic epilepsies. Although our findings cannot be generalised to all general epilepsy clinics, at present most genetic testing in epilepsy is undertaken after specialist referral and mainly in patients with developmental abnormalities and/or drug-resistant epilepsies which is in keeping with cohort that we report in this study. Due to the small sample size, we could not analyse the outcome of specific genetic epilepsies separately; as our approach was meant to assess the impact of genetic diagnosis overall. This was an observational retrospective study, and not a randomised controlled trial of the PM approach. Some patients may have had successful treatment changes before the genetic diagnosis, for example, in DS, and these have not been included in this analysis; however, the purpose of this study was to get a real-life measure of the current implications for treatment changes following a genetic diagnosis. Furthermore, a degree of uncertainty related to genetic causation is inevitable in some cases, adding a potential bias to the study.

Our observations should lead to greater caution in raising expectation in people with epilepsy, clinicians and healthcare providers about the current impact of genetic findings in epilepsy. Conversely, our findings suggest we need to continue to develop further strategies for PM treatment in genetic epilepsies, including, for example, complementary deep and multimodal phenotyping, epigenetics, environmental risk factors, standardised functional characterisation of gene variants and appropriately designed clinical trials of targeted treatments identified through functional work.

Based on the results of our real-life survey, we recommend the following actions to improve outcome following a genetic diagnosis: if there is uncertainty regarding the pathogenicity of the mutation, functional testing should be considered; medication changes, including repurposed drugs, and further diagnostic assessment, depending on underlying genetic condition, should be considered; periodic re-evaluation of the impact of genetic testing should be planned.

There has already been significant progress in this field, with expanding genetic discoveries leading to novel approaches to understand systems biology and devise or select targeted treatments,^37–39^ and there are promising fields of medicine where PM is already being successfully applied on a large scale such as in oncology. In epilepsy, genomics is becoming increasingly adopted in clinical practice and has a meaningful impact on diagnosis^40^: for it also to realise its full promise for treatment, more work is needed to understand failure of PM in clinical practice.

## Data Availability

Data are available on reasonable request. The authors confirm that the data supporting the findings of this study are available from the corresponding author, on reasonable request and subject to protocol approvals at each contributing site.
